# Massachusetts Veterinary Medical Association

**Published:** 1896-07

**Authors:** Howard P. Rogers

**Affiliations:** Secretary


Secretary.

### MASSACHUSETTS VETERINARY ASSOCIATION.

The thirteenth annual meeting was held at Young's Hotel, Boston, April 22, 1896. The only business of importance was the election of officers, with the following result: President, Dr. John M. Parker, Haverhill; First Vice-President, Dr. Madison Bunker, Newton; Second Vice-President, Dr. Daniel Emerson, Lynn; Secretary and Treasurer, Dr. Howard P. Rogers, Allston; Executive Committee, Drs. Thomas Blackwood, Boston; W. E. Peterson, Waltham; Henry Lewis, Chelsea; J. R. McLaughlin, Newton; George Lee, Brighton.

After the business was completed the members adjourned to the banquet hall. As there were no guests present, the after-dinner exercises were very informal.

Howard P. Rogers, V.S.,
Secretary.

